# Unilateral Perioral, Thumb, and Thenar Numbness Secondary to Acute Thalamic Infarct

**DOI:** 10.7759/cureus.4909

**Published:** 2019-06-16

**Authors:** Jennifer Obasi, Justin C Chen, Trenton VandeWater

**Affiliations:** 1 Internal Medicine, Medical College of Wisconsin Affiliated Hospitals, Milwaukee, USA; 2 Emergency Medicine, Medical College of Wisconsin, Milwaukee, USA; 3 Physical Medicine and Rehabilitation, Medical College of Wisconsin, Milwaukee, USA

**Keywords:** numbness, stroke, paresthesia, perioral

## Abstract

Lacunar infarcts are small, deep infarcts that occur in subcortical regions of the brain and can result in pure sensory stroke syndromes, ataxia, and dysarthria. The most common predisposing etiology is small-vessel lipohyalinosis or microatheroma formation usually secondary to diabetes mellitus or systemic hypertension. We report a patient who presented with cheiro-oral syndrome (COS) with left sided perioral, thumb, and thenar numbness after an acute lacunar infarct of the right thalamus.

## Introduction

Lacunar strokes make up approximately 15%-21% of all strokes [1-2]. These infarcts occur in the basal ganglia (including the thalamus), subcortical white matter and pons, usually affecting a singular penetrating branch of a larger cerebral artery. Blockage of these small arteries typically occurs due to lipohyalinosis and embolic or atheromatous occlusion [3]. Similar to other strokes, chronic hypertension is the most important risk factor [4]. Acute thalamic stroke can present with a variety of sensory and motor deficits based on the topographic area of infarction [5]. Less commonly, acute thalamic stroke can present with cheiro-oral syndrome (COS), characterized by purely sensory deficits in unilateral fingers and ipsilateral perioral region, with its distribution making it easy to miss in the acute setting [6].

## Case presentation

A 71-year-old man presented to ED with a chief complaint of left-sided numbness. Approximately five hours prior to arrival, the patient developed sudden onset of left-sided perioral, thumb, and thenar numbness. Past medical history was significant for coronary artery disease, type 2 diabetes, chronic kidney disease, hypertension, and clear cell renal cell carcinoma status post nephrectomy (10 years prior). The patient had taken two tablets of baby aspirin prior to arrival. He denied having any deficits prior to onset of symptoms, and denied any changes in vision, speech difficulties, weakness, ataxia, chest pain, palpitations, or shortness of breath. Vitals were notable for hypertension with systolic blood pressures ranging from 150s to 190s. Symptoms resolved spontaneously about 30 min after arrival to the ED and on examination the patient was found to have no residual neurological deficits. The patient denied secondary causes for perioral numbness including hypocalcemia, thyroid dysfunction, history of seizures, and/or migraines. Due to complaint of lateralized paresthesias and multiple risk factors for stroke, workup for a potential transient ischemic attack was begun.

**Figure 1:**
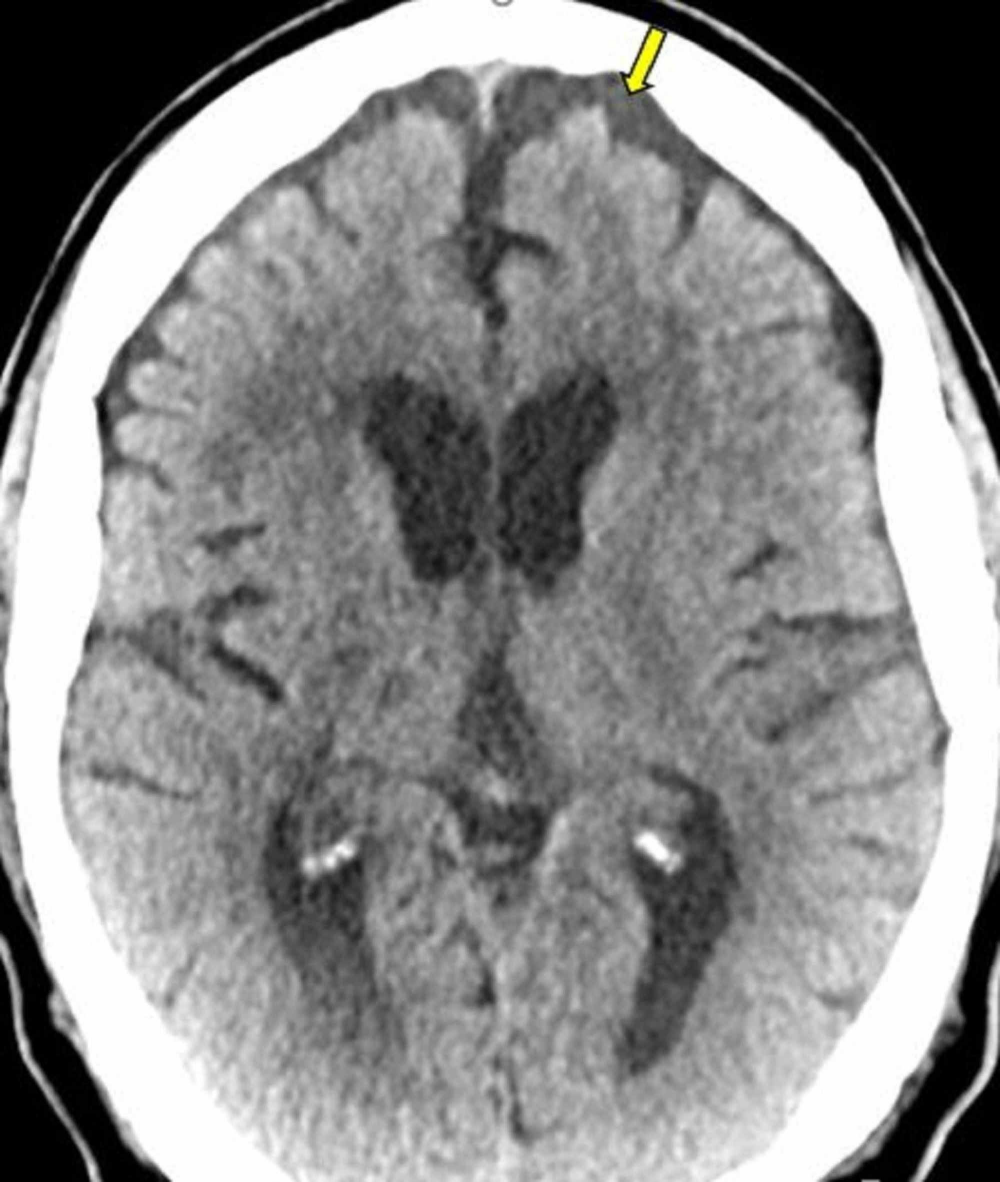
CT of the head showing age appropriate involutional changes (yellow arrows). No acute intracranial process.

Laboratory workup was significant for serum creatinine of 1.28 mg/dL and glucose of 213 mg/dL, otherwise basic metabolic panel (BMP) was normal. Thyroid stimulating hormone (TSH), complete blood count (CBC), and coagulation screen were unremarkable. Lipid panel was remarkable for cholesterol of 199 mg/dL, low density lipoprotein (LDL) of 98 mg/dL, triglyceride of 259 mg/dL, and high density lipoprotein (HDL) of 49 mg/dL. Glycated hemoglobin was 7.0%. Initial imaging was done with noncontrast CT of the head which showed age-appropriate involutional changes without acute intracranial hemorrhage (Figure 1). 

Computed tomography angiography (CTA) of the head and neck was unremarkable. Transthoracic echocardiogram (TTE) did neither reveal a patent foramen ovale nor thrombi. Electrocardiogram (ECG) was unchanged from prior and revealed normal sinus rhythm.

**Figure 2:**
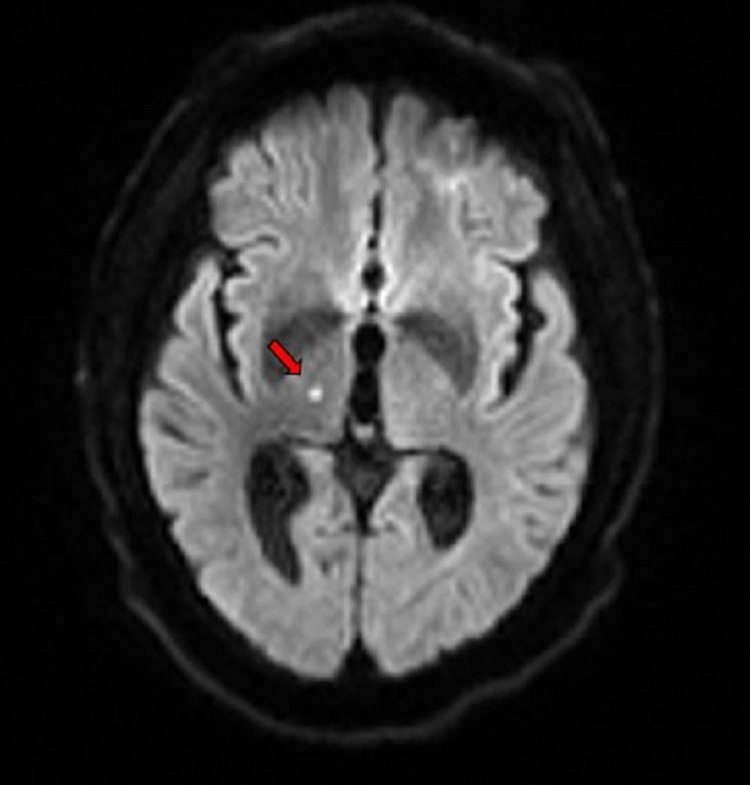
MRI of the brain showing punctuate foci of restricted diffusion in the right thalamus (red arrow) consistent with an acute lacunar infarct.

**Figure 3:**
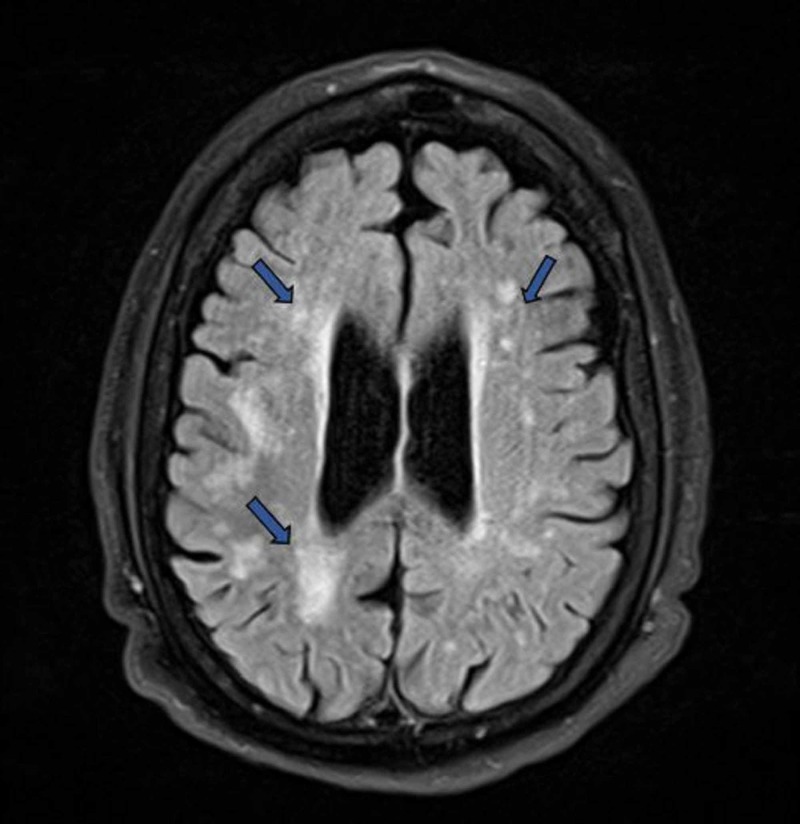
MRI of the brain showing multifocal and confluent hyperintense lesions in the periventricular white matter (blue arrows), most likely due to chronic small vessel ischemia.

Follow up imaging was done with MRI of the brain which showed an acute lacunar infarct of the right thalamus and other chronic changes (Figures 2-3).

The patient was diagnosed with an acute right thalamic infarct with findings consistent with COS. He was not a candidate for thrombolytic therapy with intravenous alteplase. He was started on high-intensity statin therapy, and dual antiplatelet therapy (DAPT) with aspirin and clopidogrel for 30 days, with plans to continue clopidogrel monotherapy thereafter. His antihypertensive medication regimen was also optimized, and he was advised to quit smoking.

## Discussion

Cheiro-oral syndrome is an acute stroke syndrome with a variety of sensory impairments based on distribution. Etiologies include ischemia, hemorrhage, tumor, or vascular malformation [7]. Ischemia and hemorrhage are the leading causes [8]. The most common site of infarcts presenting as COS include the pons and thalamus [8]. A prospective study done to delineate the localization and pathogenesis of COS classified four subtypes of COS based on distribution of sensory impairments. Type 1 COS was a sensory impairment limited to the perioral area and homolateral finger(s)/hand in unilaterality. Type 2 COS was a sensory impairment limited to the perioral area and finger(s)/hand in bilaterality. Type 3 COS was a sensory impairment limited to the perioral area and finger(s)/hand where one is involved in bilaterality and the other unilaterality. Type 4 COS was a sensory impairment limited to the perioral area and opposite finger(s)/hand, or crossed COS [8]. Diabetes mellitus and hypertension are common risk factors. Noncontrast CT of the head is the first diagnostic imaging study done in patients with suspected acute stroke, but its sensitivity for detecting lacunar infarcts is low. If clinical suspicion is high, this should be followed up with MRI of the brain [9]. Pure sensory strokes, without associated motor deficits are found in up to 18% of lacunar stroke syndromes [8], although the true prevalence may be underestimated, especially in patients with transient ischemic attack (TIA).

## Conclusions

Hypertension is a common predisposing condition in patients diagnosed with lacunar infarcts as was evident in the patient presented who had a longstanding history of poorly controlled hypertension. The patient also had a history of diabetes and 50 pack-year smoking history which also increased his risk for cerebrovascular disease.
